# Self-Powered Photodetectors with Ultra-Broad Spectral Response and Thermal Stability for Broadband, Energy Efficient Wearable Sensing and Optoelectronics

**DOI:** 10.3390/molecules30142897

**Published:** 2025-07-08

**Authors:** Peter X. Feng, Elluz Pacheco Cabrera, Jin Chu, Badi Zhou, Soraya Y. Flores, Xiaoyan Peng, Yiming Li, Liz M. Diaz-Vazquez, Andrew F. Zhou

**Affiliations:** 1 Department of Physics, University of Puerto Rico, San Juan, PR 00936, USA; elluz.pacheco@upr.edu (E.P.C.); soraya.flores@upr.edu (S.Y.F.); yiming.li@upr.edu (Y.L.); 2 Chongqing Key Laboratory of Brain-Inspired Computing and Intelligent Control, Southwest University, Chongqing 400715, China; chujin@swu.edu.cn (J.C.); pengxy2015@swu.edu.cn (X.P.); 3 Department of Chemistry, Biochemistry, and Physics, Indiana University of Pennsylvania, Indiana, PA 15705, USA; badi.zhou@pm.me; 4 Department of Chemistry, University of Puerto Rico, San Juan, PR 00936, USA; liz.diaz2@upr.edu

**Keywords:** two-dimensional boron nitride (BN) nanosheets, plasmonic gold nanoparticles, self-powered photodetector, ultra-broadband DUV to MIR response, photovoltaic mode, photoconductive mode, p-n heterojunction, plasmon-enhanced absorption, thermal stability, wearable sensing and optoelectronics

## Abstract

This work presents a high-performance novel photodetector based on two-dimensional boron nitride (BN) nanosheets functionalized with gold nanoparticles (Au NPs), offering ultra-broadband photoresponse from 0.25 to 5.9 μm. Operating in both photovoltaic and photoconductive modes, the device features rapid response times (<0.5 ms), high responsivity (up to 1015 mA/W at 250 nm and 2.5 V bias), and thermal stability up to 100 °C. The synthesis process involved CO_2_ laser exfoliation of hexagonal boron nitride, followed by gold NP deposition via RF sputtering and thermal annealing. Structural and compositional analyses confirmed the formation of a three-dimensional network of atomically thin BN nanosheets decorated with uniformly distributed gold nanoparticles. This architecture facilitates plasmon-enhanced absorption and efficient charge separation via heterojunction interfaces, significantly boosting photocurrent generation across the deep ultraviolet (DUV), visible, near-infrared (NIR), and mid-infrared (MIR) spectral regions. First-principles calculations support the observed broadband response, confirming bandgap narrowing induced by defects in h-BN and functionalization by gold nanoparticles. The device’s self-driven operation, wide spectral response, and durability under elevated temperatures underscore its strong potential for next-generation broadband, self-powered, and wearable sensing and optoelectronic applications.

## 1. Introduction

Two-dimensional (2D) boron nitride (BN) nanosheets have garnered significant attention due to their unique atomic structure and exceptional mechanical, chemical, electrical, thermal, and optoelectronic properties. These attributes enable diverse applications, including spintronics, tissue engineering, catalysis, deep ultraviolet (DUV) light emitters, and critical components in nanodevices [1,2,3,4,5]. Compared to traditional semiconductors, BN stands out for its superb thermal stability and chemical inertness, making it ideal for harsh-environmental devices. For example, recent advances have yielded visible-blind BN photodetectors operational at 400 °C [6].

Various techniques have been developed for BN synthesis, including plasma sputtering [7], chemical vapor deposition (CVD) [8,9], thermal vapor solid target [10], chemical blowing [11], ball milling process [12], micromechanical cleavage [13], and exfoliation [14,15] of bulk hexagonal boron nitride (h-BN). More recently, scalable production has been achieved through bottom-up approaches [16] and salt-template methods [17], enabling precise control over sheet thickness, edge structures, and functionalization. This tunability is critical: functionalization or edge engineering can modulate the bandgap of 2D BN, which governs its electrical properties. For instance, pulsed laser plasma deposition has been used to fabricate atomically thin BN sheets for low-leakage diodes [18] and BN-graphene heterostructures for charge tunneling studies in high-frequency transistors [19]. Notably, plasma treatment of atomic-thin BN sheets induces an 8% red shift of the cut-off wavelength from 220 nm to 250 nm [20].

As a wide bandgap semiconductor (5.97 eV), bulk BN is a promising candidate for deep ultraviolet (DUV) detection without solar rejection filters. BN-based photodetectors exhibit solar-blind operation, high gain, and signal-to-noise ratios. Wang et al. grew BN films on sapphire with a sharp cut-off at 220 nm [15], while Zhang et al. develop atomically thin hBN photodetectors via heterostructures [21]. Most studies focus on visible-blind DUV photodetectors with cut-offs below 230 nm [22,23,24,25], and a recent review has outlined their progress and challenges [26]. Beyond DUV sensing, h-BN has shown potential in wearable technologies for breath monitoring [27], glucose sensing [28], and strain/pressure-sensitive skin sensors [29], highlighting its versatility in flexible electronics.

Theoretical studies predict that 2D hetero-nanostructures outperform single-material devices via synergistic effects [30,31]. Several efforts have focused on BN energy bandgap modulation techniques based on binary structures, showing potential for heterostructure tunneling devices and broadband photodetection. BN-tungsten nitride (WN) heterostructures, for example, enable tunable bandgaps for UVB detection up to 360 nm [32]. Two-dimensional BN-analogous materials like graphene and layered transition metal dichalcogenides (TMDs, e.g., MoS_2_, WS_2_, WSe_2_, and MoSe_2_) have also been explored for their exotic properties [20,33,34,35,36].

Despite progress, BN-based ultra-broadband photodetectors remain underexplored. Most broadband photodetectors rely on graphene- or oxide-based semiconductors [37,38,39,40,41], which suffer thermal instability at elevated temperatures. In contrast, BN-based detectors appeared to have better stability above 250 °C. Here, we report a super broadband (0.2–5 eV) photodetector using Au nanoparticle (NP)—functionalized 2D BN nanosheets. Unlike traditional DUV photodetectors requiring external power, this self-powered design reduces size, cost, and energy consumption, making it ideal for wide-spectrum monitoring in hazardous environments.

## 2. Photodetector Characterizations

### 2.1. SEM and TEM Images

**Figure 1:**
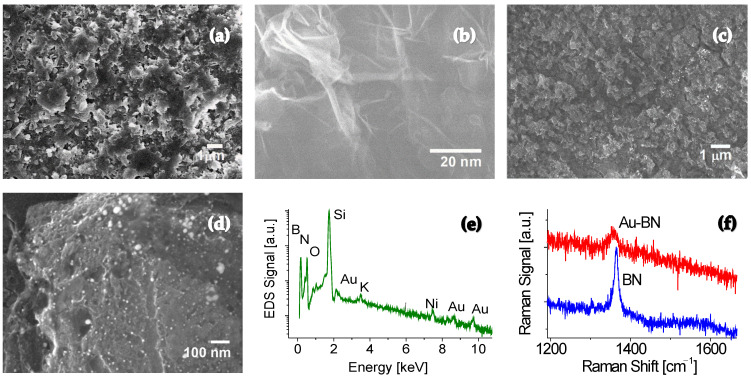
Characterizations of 2D BN sheets before and after Au NP treatment. (**a**) SEM and (**b**) TEM images of a pristine BN sample with different magnifications. (**c**,**d**) SEM images of the sample after Au-NPs functionalization. (**e**) EDS and (**f**) Raman spectra of Au-BN sample.

Scanning electron microscope (SEM) analysis was conducted to examine the surface morphologies of 2D BN nanosheets. Figure 1a shows typical SEM images of a pristine BN sample prepared on a Si substrate before Au NPs treatment. The sample appears to be 3D architectures composed of multiple overlapping 2D nanosheets in random orientations, with an average sheet size of around 1 µm × 1 µm. Figure 1b presents transmission electron microscope (TEM) images of different magnifications. The slightly dark regions likely represent overlapping or ripple areas, visually confirming the presence of multiple 2D BN nanosheets.

Further structural analysis using high-resolution TEM (HRTEM) revealed the characteristic honeycomb lattice of hBN on the surface region, along with approximately 2 to 10 stacked atomic layers observed at the edge area. The synthesized sample comprises multiple BN nanosheets arranged in random orientations, with minimal defects [5]. Figure 1c,d illustrate SEM images of BN nanosheets following Au nanoparticle treatment. The overall 3D architecture of the nanosheets remains unchanged. The higher-magnification SEM image in Figure 1d reveals a uniform yet dispersed distribution of Au nanoparticles across the nanosheet surfaces. The average particle size is approximately 7 nm, with an estimated density of ~35 particles per 100 × 100 nm^2^ area.

### 2.2. EDS Measurements

The composition of the Au functionalized BN sample was analyzed using energy-dispersive X-ray spectroscopy (EDS), as shown in Figure 1e. The peaks at 0.177 keV and 0.41 keV belong to B and N, respectively, indicating the successful synthesis of BN. Low concentrations of carbon and oxygen impurities were also detected, likely originating from the contaminated chamber and residue gas in the chamber during the synthesis. The peaks at binding energies of 2.22 keV, 8.6 keV, and 9.72 keV correspond to Au, whereas the peak at 1.734 keV corresponds to Si from the substrate, which has the highest peak among all the elements detected in the Au functionalized BN nanosheet sample.

### 2.3. Raman Spectroscopy

Raman spectroscopy was performed to analyze the sample before and after Au NP treatment, with the data presented in Figure 1f. The Raman peak at 1362 cm^−1^ from the untreated BN sample corresponds to the active E_2g_ vibrational mode of BN [5,6]. The narrow Raman spectrum with a full width at half maximum (FWHM) of 12.7 cm^−1^ suggests that the present deposition technique produces high-quality BN composites. Background noise is attributed to a short accumulation time and extremely thin nature of the 2D BN sheets. Increasing the laser power and slowing the sweep speed in the measurements can improve the signal-to-noise ratio (SNR).

New spectral features emerge in the Raman spectrum of Au-treated BN sheets. The Raman active E_2g_ mode with the hexagonal phase shifts from 1362 cm^−1^ for the untreated BN to 1355 cm^−1^ for the Au NP functionalized BN sheets. In addition, a slightly asymmetric profile and a significantly broadened band with an FWHM up to 31.3 cm^−1^ are clearly visible, providing solid evidence of successful surface functionalization of the 2D BN sheets.

### 2.4. Current-Voltage (I-V) Electric Characterization

**Figure 2:**
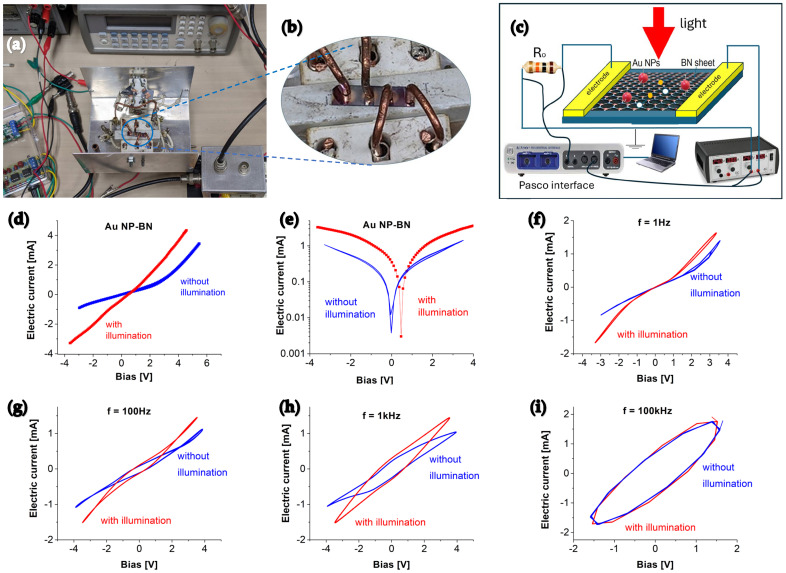
(**a**) Schematic illustration of the measurement configuration. (**b**) Experimental setup. (**c**) Photograph of the fabricated Au functionalized BN photodetector. (**d**) Linear and (**e**) semilog plots of the I-V characteristics of photodetector under dark and blue light illumination. I-V responses under AC bias at frequency of (**f**) 1 Hz, (**g**) 100 Hz, (**h**) 1 kHz, and (**i**) 100 kHz.

After the basic characterizations were completed, the prototype was fabricated, where a gold Schottky contact at each end of the sample was made to form a self-driven photodetector, as shown in Figure 2a,b. A functionalized BN-based membrane with a thickness of around ~1 µm was used as an active layer of the prototype. The total exposure area of the active region was 0.5 × 7 mm^2^. Following annealing at 800 °C for 30 min, the prototype was electrically studied first by using a PASCO 850 universal interface and an HP34401 multimeter, and then followed by characterizations of the responsivity to super broadband light illuminations.

A schematic illustration of the measurement configuration is given in Figure 2c. All measurements were carried out under standard ambient conditions without and with light illumination. Figure 2d,e shows the typical I-V characteristics of the prototype operating at room temperature with and without 10 mW/cm^2^ blue light (λ = 450 nm) irradiation. The complete I-V curve exhibits a typical behavior of a simple PN junction diode, which conducts electric current only in one direction. Light illumination clearly created a noticeable change in the original IV curve. This alteration is attributed to photo-induced electron-hole pairs within the depletion region in the interface, which results in a decrease of p-n junction barriers and a modification of the device’s electrical behavior.

Stable hysteresis loops were obtained in the heterostructure under AC bias conditions as shown in Figure 2f–i. When an AC power supply is applied, the conditions for the Schottky barrier are disrupted, particularly due to thermal effects. The hysteresis is possibly associated with the presence of trap states at the Schottky contact interface. These traps can capture and release electrons, influencing the current flow through the diode. The density and distribution of these interface trap states are influenced by the quality of the metal-semiconductor interface. The observed phenomena probably suggest resistive switching behavior, due to interfacial properties and intrinsic defects within the bilayer structure. The non-zero open-circuit voltage increases with the bias frequency when the dark current is zero. Similar phenomena are also observed for the short-circuit current at zero bias voltage.

Under blue light illumination, photons with energies exceeding the bandgap generate electron-hole pairs, leading to an increase in carrier density and enhanced conductivity, which accounts for the higher current observed in the hysteresis curves. However, in the region near the open circuit voltage or short circuit current, the hysteresis remains nearly unchanged regardless of light illumination, suggesting limited photoresponse in those bias ranges. A larger hysteresis loop area is observed at a higher bias frequency, but no significant light-induced photocurrent is detected. Comparable phenomena have been reported in oxide and nitride semiconductors, supporting the interpretation of the observed hysteresis effect [42,43].

### 2.5. Spectral Response to Different Illumination Wavelengths

**Figure 3:**
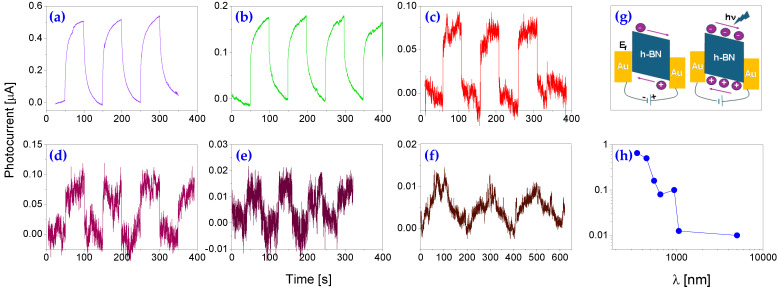
Cyclic photoresponse measurements of the prototype under 1 mW/cm^2^ illumination at wavelengths of (**a**) 450 nm, (**b**) 550 nm, (**c**) 650 nm, (**d**) 940 nm, (**e**) 1064 nm, and (**f**) 5900 nm, conducted at a bias voltage of 0.5 V and room temperature. (**g**) Schematic illustration of the underlying detection mechanism. (**h**) Spectral response of the device as a function of wavelength. E_F_ represents the Fermi level in the h-BN semiconductor.

As the first test, the photoconductive metal–semiconductor–metal (MSM) mode was used because of its simplicity and low-cost device design. Figure 3 shows cyclic tests operating at room temperature and under a 0.5 V bias. The light-induced photocurrents exhibit desirable features such as a stable baseline and repeatability. A strong response under infrared illumination is clearly observed, indicating a significant extension of the cut-off wavelength compared to conventional bulk BN-based photodetectors, which typically exhibit a sharp cut-off near 230 nm.

As seen in the cyclic tests under 450 nm blue light radiation (Figure 3a), the induced photocurrent rises rapidly at first and then gradually reaches its maximum. Once the light is turned off, the photocurrent quickly drops. The induced photocurrent is closely associated with light absorption. From the obtained data, the peak photocurrent under blue light is around 0.51 µA. Since the exposed surface area of the prototype is around 3.5 mm^2^ and the light intensity on the surface is 1 mW/cm^2^, correspondingly, the responsivity (induced photocurrent to light power) is estimated to be around 14.33 mA/W. A similar trend is observed at longer wavelengths under the same light intensity. For instance, green light (λ = 550 nm, Figure 3b) generated a photocurrent of 165 nA, whereas red (λ = 650 nm, Figure 3c), near-infrared (NIR, λ = 940 nm and 1064 nm, Figure 3d,e), and mid-infrared (MIR, λ = 5900 nm, Figure 3f) light produced photocurrents of 75, 90, 11, and 8.2 nA, respectively.

In the absence of light, as shown in Figure 3g, thermal energy alone is insufficient to generate a significant number of charge carriers, resulting in minimal dark current. Upon illumination (hν), electron–hole pairs are generated in the h-BN structure, with electrons moving toward one Au electrode and holes toward the opposite one, indicating effective photogenerated charge separation. This leads to a measurable photocurrent through the circuit. Overall, as illustrated in Figure 3h, the photocurrent decreases gradually with increasing wavelength from UV to red, followed by a slight increase in the NIR range. A similar trend was observed under zero-bias conditions, which will be discussed later.

As shown in Figure 3, the photodetector demonstrates a broadband response ranging from deep UV to NIR and MIR. While the data suggest potential sensitivity extending into the terahertz (THz) range, experimental validation was constrained by the limited availability of suitable radiation sources. In general, the broadband spectral response is attributed to both the intrinsic properties of the nanocomposite and the complex interplay of multiple contributing factors. These underlying mechanisms are further discussed in the Section 3.

### 2.6. Self-Powered Photodetector Operating in Photovoltaic Mode at Zero Bias

**Figure 4:**
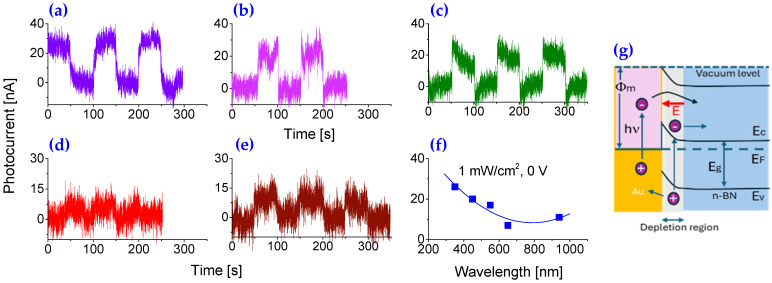
Cyclic photoresponse under 1 mW/cm^2^ illumination at wavelengths of (**a**) 365 nm; (**b**) 450 nm; (**c**) 550 nm; (**d**) 650 nm; and (**e**) 940 nm, performed at zero bias (0 V) and room temperature. (**f**) Spectral response of the device as a function of wavelength at zero bias. (**g**) Schematic illustration of the detection mechanism operating in photovoltaic mode where Φₘ represents the work function of the metal (Au), which is the energy difference between the vacuum level and the Fermi level of Au, E_c_, E_v_, E_g_ and E_F_ represent the conduction band edge, valence band edge, bandgap and Fermi level, respectively, in the n-BN semiconductor.

In metal–semiconductor–metal (MSM) photodetectors, one drawback is that the long carrier lifetime can significantly reduce response speed. Moreover, the need for an external power supply increases the system’s size, cost, and energy consumption, limiting its suitability for continuous, long-term monitoring, particularly in hazardous environments. By incorporating the structure of Schottky barrier and p-n heterojunction, as well as its unique properties, the fabricated photodetector can be operated in photovoltaic mode. Au functionalized 2D BN nanosheets enable the prototype to operate in a self-driven, highly sensitive mode with an ultra-broad spectrum response range from 0.2–5.9 μm. Similar to Figure 3, in related experiments, different wavelengths of light have been applied, whereas their light intensities remain unchanged. Figure 4a–e shows the variations of the induced photocurrents from the prototype exposed to different light of wavelengths at 365 nm, 450 nm, 550 nm, 650 nm, and 940 nm illuminations during the cyclic test with a period of 100 s between the “switch-on” and “switch-off” at light intensity of 1 mW/cm^2^.

When the prototype is exposed to 365 nm UV light (Figure 4a), the induced photocurrent quickly rises at first and then reaches a stable value. When the UV light is turned off, the light-induced photocurrent decreases quickly and then gradually decays to its original state. The obtained maximum photocurrent is 26 nA, and the dark current is 4 nA, yielding an SNR up to 6. Since light intensity is 1 mW/cm^2^ and the exposed area of the active layer is 3.5 mm^2^, the estimated responsivity of the prototype, R, can be obtained around 0.8 mA/W. This value is almost two orders larger than that obtained from pristine BNs based [5,6], and comparable to what has been recently reported on oxide semiconductor-based visible-blind UV photodetectors [44,45].

As a comparison, the detector’s responses to blue, green, red, and infrared light were also measured, with the results presented in Figure 4b–e, respectively. Clearly, the response to 940 nm infrared light was significantly weaker, around half the photocurrent yielded under 360 nm or 450 nm illuminations at the same intensity, indicating the fabricated BN nanosheet-based detector is more sensitive to UV and visible wavelengths. Although the induced photocurrent under 940 nm illumination was relatively low and accompanied by a poor SNR, it remained detectable, as shown in Figure 4e. Importantly, no sharp cut-off in photoresponse was observed (Figure 4f), which contrasts with previous reports on 2D BN nanosheets-based deep UV photodetectors that demonstrated a distinct visible-blind cut-off [15,24,25,26]. Furthermore, the excellent performance of the detector under investigation in the deep UV regions remains nearly unchanged.

The schematic given in Figure 4g shows the energy bands before and after aligned at the interface between the gold (Au) and n-type boron nitride (n-BN), depicting the detection mechanism under photovoltaic mode where Φₘ represents the work function of the metal (Au), which is the energy difference between the vacuum level and the Fermi level of Au, E_c_, E_v_, and E_F_ represent the conduction band edge, valence band edge, and Fermi level, respectively, in the n-BN semiconductor. The band bending in the n-BN near the interface forms a Schottky barrier, which leads to the formation of a depletion region (indicated at the interface). When the photodetector is illuminated with photons of energy hν ≥ E_g_ (bandgap of n-BN), electron–hole pairs are generated. The built-in electric field in the depletion region drives the electrons toward the n-BN and the holes toward the Au electrode. This separation of photogenerated carriers under zero external bias results in a photocurrent, enabling self-powered (photovoltaic) operation. The red arrow labeled E indicates the movement of the photogenerated electron under the influence of the internal electric field. The figure also shows carrier excitation and transport across the junction, illustrating how light energy is converted into an electrical signal.

### 2.7. Bias Effect on the Induced Photocurrent at Visible and UV Wavelengths

**Figure 5:**
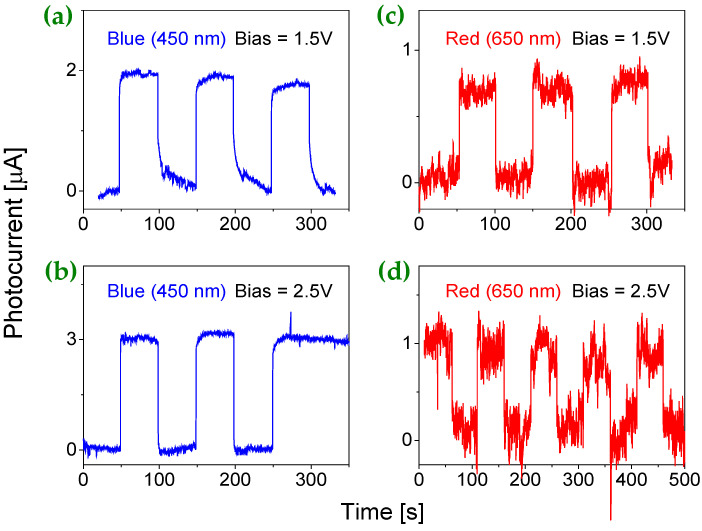
The responses at bias voltages of 1.5 V and 2.5 V under an illumination intensity of 1 mW/cm^2^ and RT, shown for (**a**,**b**) λ = 450 nm (blue) and (**c**,**d**) λ = 650 nm (red).

Before characterizing the prototype in a photovoltaic mode, the effect of bias voltage on light-induced photocurrent was studied using a planar metal-semiconductor-metal (MSM) structure with a simple geometry suitable for photodetector applications. Experimental results indicate that the increase of bias voltage efficiently enhances the yield of the induced photocurrent. Figure 5 shows the induced photocurrents by blue (Figure 5a,b) and red light (Figure 5c,d) as a function of bias for the prototype operated at room temperature (RT) and radiation intensity of 1 mW/cm^2^. A higher bias yields a higher photocurrent or a larger responsivity R (defined as the photocurrent output relative to the incident light input). For red light, the induced photocurrents at bias voltages of 1.5 V and 2.5 V are about 10 and 16 times that at a bias voltage of 0.5 V.

At the same bias voltage magnitudes, the blue light induced photocurrents show increases of about 4 and 6 times, respectively. These phenomena can be directly attributed to the MSM structure, where the current is primarily carried by the majority charge carriers. The flow of majority carriers is strongly influenced by the accumulation of minority carriers at one of the contacts, which in turn promotes additional injection of majority carriers until recombination occurs. This process leads to a high photoconductive gain. However, a major limitation of this configuration is the large dark current caused by the high bias voltage, which consequently lowers the signal-to-noise ratio.

**Figure 6:**
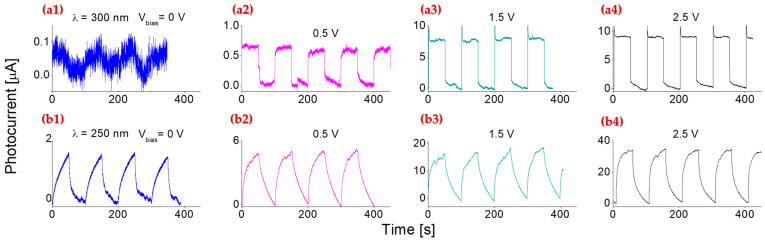
Room temperature cyclic response tests of the detector under 1 mW/cm^2^ illumination at 300 nm (**a1**–**a4**) and 250 nm (**b1**–**b4**) with bias voltages of 0 V, 0.5 V, 1.5 V, and 2.5 V, respectively.

**Figure 7:**
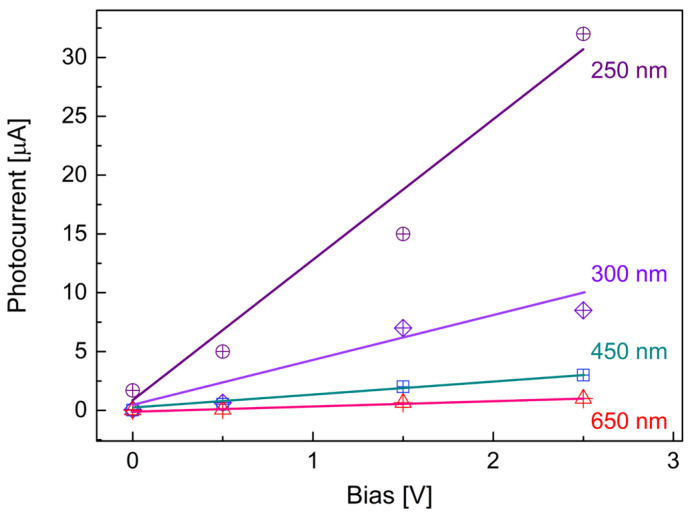
Room temperature photoresponses of the detector under 1 mW/cm^2^ of illumination at wavelengths of 250, 300, 450, and 650 nm, with bias voltages varied from 0 to 0.5, 1.5, and 2.5 V. The solid lines represent linear fits to the corresponding measured data points at each wavelength.

Figure 6 shows the response of the detector when exposed to 1 mW/cm^2^ deep UV illumination at different biases. The cyclic testing data exhibit a stable baseline and clear response signals with high reproducibility. The maximum photocurrents obtained were around 1.7, 5, 15, and 32 µA when exposed to 1 mW/cm^2^ of 250 nm light illumination at bias voltages of 0, 0.5, 1.5, and 2.5 V, respectively. In photoconductive mode, the SNR of the photoresponse was evaluated by comparing the photocurrent amplitude with the dark current fluctuations (Figure 6(a2–a4,b2–b4)). All illuminated signals exhibit an SNR > 10^3^, confirming that the observed responses are dominated by photoexcitation rather than noise. Figure 7 displays the photoresponse of the prototype to deep UV illuminations with an intensity of 1 mW/cm^2^, along with responses to visible light at 450 and 650 nm under the same illumination conditions as the bias voltage was increased from 0 to 2.5 V.

Compared with previous studies [5,6,30,32], the following four key differences are observed:

(1) The photocurrent of 1.7 mA generated from the self-driven prototype under 250 nm UV illumination shows a significant enhancement, at least an order of magnitude larger than that of a self-driven BN/WN based prototype (0.84 mA) [32], and approximately three orders of magnitude greater than the previously reported unfunctionalized 2D BN nanosheet based photodetectors (0.15 nA) [5].

(2) At the same operating conditions, 250 nm light-induced photocurrent under the investigation is approximately 10, 8, 5, and 3 times higher than that induced by 300 nm light at the biases of 0, 0.5, 1.5, and 2.5 V, respectively.

(3) While higher bias voltages generally result in increased photocurrent, the rate of photocurrent increase is more pronounced for shorter-wavelength (deep UV) illumination compared to longer-wavelength (visible) light.

(4) Under 300 nm illumination, both the response and recovery times were consistently fast, on the order of a few milliseconds, and remained stable throughout repeated cycles. In contrast, the 250 nm included photocurrent exhibited longer response and recovery times. Interestingly, plasma-treated 2D BN nanosheet-based photodetectors reported the opposite trend, where 250 nm illumination exhibited faster dynamics than 300 nm light [27]. This discrepancy is likely associated with variations in the band structures.

### 2.8. Thermal Effect on the Induced Photocurrent

Besides the bias effect, experiments were also carried out to investigate the influence of temperature on the prototype’s performance. Several research groups have previously reported their progress in developing high-temperature DUV photodetectors based on multilayered oxide semiconductors [40,41] and SiC-based material [46]. However, these reported detectors typically failed to operate effectively above 200 °C [20]. In contrast, BN-based detectors appeared to have superior thermal stability at high temperatures. When the temperature changed from 20 to 150 °C, the deep UV light-induced photocurrent remained nearly constant. At 250 °C, strong thermal noise began to appear in the 250 nm induced photocurrent, whereas 300 nm and 365 nm induced photocurrents remained stable. At 400 °C, strong thermal noise was observed in the 350 nm induced photocurrent, and the 250 nm light-induced photocurrent became very weak and difficult to distinguish from background noise, resulting in a poor signal-to-noise ratio. Meanwhile, the 300 nm induced photocurrent remained unchanged [27].

**Figure 8:**
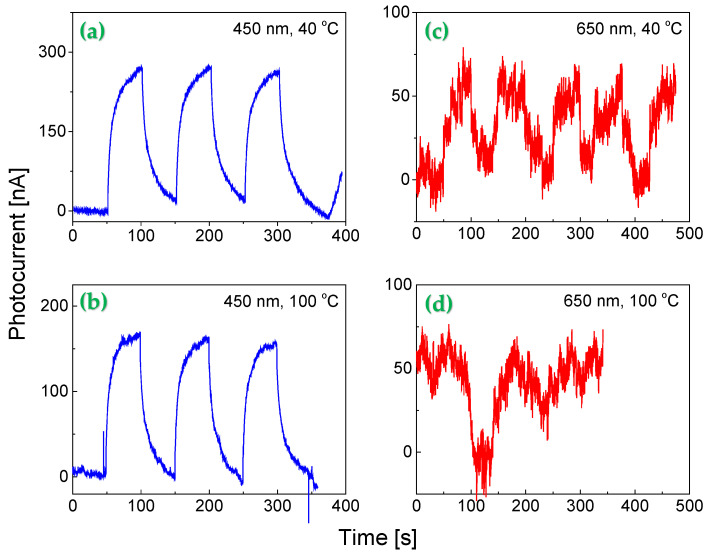
Thermal effect on the photoresponse of the prototype under 1 mW/cm^2^ illumination at a bias voltage of 0.5 V. Response to (**a**,**b**) 450 nm (blue) and (**c**,**d**) 650 nm (red) light at 40 °C and 100 °C, respectively.

To determine whether these features hold for the present study, the thermal effect on the response of the Au NP 2D BN nanosheet-based prototype was evaluated under 1 mW/cm^2^ red and blue light illuminations. As shown in Figure 8, variations in operating temperature significantly affected the performance of the prototype. The strength of this influence closely relies on the composite structure of active layers and the incident light wavelength.

The 450 nm blue light induced photocurrent remained relatively stable with an increase in operating temperature from 40 °C to 100 °C, as the induced photocurrent slightly decreased from 250 nA to 170 nA with a minimal increase in background noise (Figure 8a,b). The signal-to-noise ratio up to 30 remained nearly unaffected by temperature increase. In contrast, the 650 nm red light induced photocurrent had a much stronger noise background (Figure 8c). The peak photocurrent dropped down to 50 nA and then further decreased with an increase in operating temperature (Figure 8d), where the weak photocurrent is largely masked by strong background noise, resulting in a poor signal-to-noise ratio. The findings suggest that thermal effects have a more pronounced impact on the photocurrent induced by longer wavelengths (red) than by shorter wavelengths (blue or UV) light.

One notable advantage of the present Au NP functionalized 2D BN nanosheet-based prototype is its ultrabroadband photoresponse that comes at the cost of reduced signal-to-noise ratio, especially in the long wavelength region. Generally, Au NPs related junctions are an effective way to enhance the photocurrent generation. However, these Au NPs related junctions are highly susceptible to thermal disturbances. As temperature rises, the random movement of electrons within a conductor becomes more intense, leading to elevated thermal noise. Simultaneously, the kinetic energy of the nanoparticles on the surface of 2D BN sheets increases, granting them greater mobility. As a result, nanoparticles will have greater freedom to move. This will inevitably disturb Au NPs related electronic structures on the surface of BN nanosheets, resulting in a weak light-induced photocurrent and poor signal-to-noise ratio of responsivity. Further studies related to the control of sizes of nanoparticles and their number concentration, as well as ingredients are necessary in order to achieve a super broad band response, while not to sacrificing too much in signal-to-noise ratio of responsivity.

### 2.9. Effect of Light Intensity on the Induced Photocurrent

**Figure 9:**
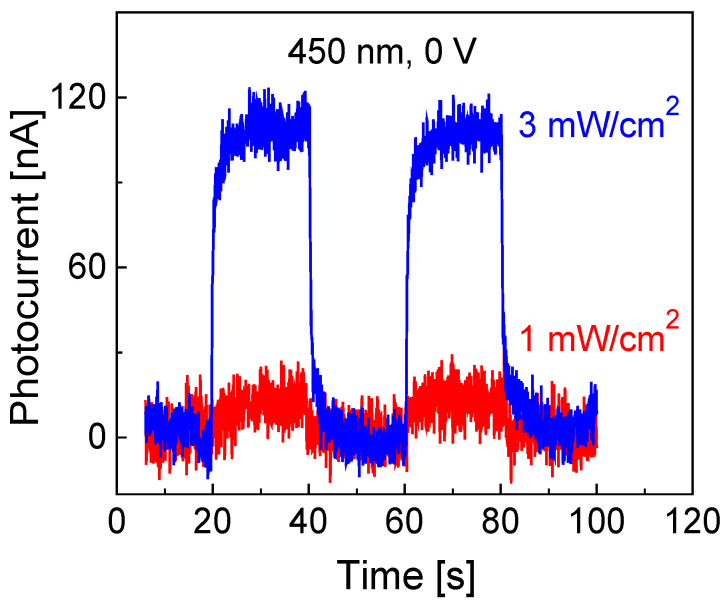
Cyclic responses under 450 nm blue light illumination at 0 V bias voltage with light intensities of 1 and 3 mW/cm^2^.

The effect of light intensity on the induced photocurrent was investigated. Figure 9 shows two typical prototype responses under different illumination intensities. The device was operated with a 40-s period at 50% duty cycle under 450 nm blue light. The prototype again exhibited good repeatability and stability. Light intensity, calibrated using an energy meter (FieldBest, Shenzhen, China), was adjusted by varying the distance between the active layer surface and the light source. An initial power density of 1 mW/cm^2^ was employed, generating a photocurrent of approximately 20 nA, as shown in Figure 9. This result is consistent with the data shown in Figure 4b.

As a comparison, the response of the fabricated prototype exposed to a power density of 3 mW/cm^2^ was also tested, with the obtained results presented in Figure 9. The induced photocurrent reached approximately 110 nA, almost 5 times as large as that obtained under a light intensity of 1 mW/cm^2^. This enhancement, which suggests the device has not yet reached saturation, is likely attributed to the material’s intrinsic electronic properties, such as its bandgap width and band structure.

### 2.10. Time Response

The time response is normally governed by the minority carrier lifetime. Previous studies have already indicated that a longer minority carrier lifetime resulted in photocurrent enhancement but at the cost of slower response speed [5,47,48]. This reflects a basic trade-off between the induced photocurrent strength and detector response speed. Usually, the minority carrier lifetime in the active layer relies on the capture rates of holes and electrons at recombination centers, which may, in turn, be influenced by the size and number density of the Au nanoparticles.

The theoretical speed limit of a photodetector depends on the transit time of electrons and holes in the device, carrier diffusion, and carrier multiplication process, as well as the circuit time constant. The response and recovery times can be directly estimated from cyclic measurements, but this method normally underestimates the actual response speed. One of the reasons is the delay in reaching full illumination intensity after turning on the lamp, and the residual photoluminescence of the light source after turning off the lamp. It can be expected that the real response and recovery times are likely much shorter than they appear. This is further supported by the role of Au NPs on the 2D BN nanosheets, which reduces the carrier recombination and then enhances the response speed.

**Figure 10:**
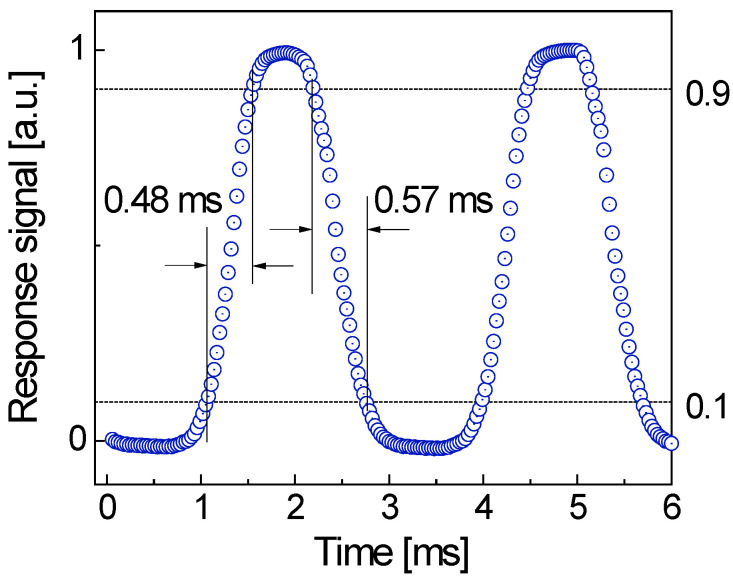
Response time and recovery time of 1 mW/cm^2^ blue light-induced photocurrent at 0.5 V bias obtained by using PASCO 850 Universal Interface together with a chopper controller.

In order to precisely analyze response and recovery times, a PASCO 850 Universal Interface (Roseville, CA, USA), together with a chopper controller (Thorlabs Inc., Newton, NJ, USA), was utilized. Representative data shown in Figure 10 indicate that the response and recovery times are less than 0.48 ms and 0.57 ms, respectively. Here, the response and recovery times are defined as the time intervals from 10% to 90% on the rising and falling edges (Figure 10). These values are much shorter than those reported for many BN-based UV photodetectors, which normally are longer than 5 ms [21].

## 3. Discussion

### 3.1. Sensing Mechanism

The performance of the photodetector is closely related to the electronic structure of the active layers and their interfaces. To precisely determine the electronic structures of Au NP functionalized 2D BN nanosheets remains a significant challenge. Even in the case of pristine 2D BN nanosheets, a lack of consensus within the field remains due to the complex interplay of interlayer positioning and the number of stacked monolayers interacting through weak Van der Waals and electrostatic forces [3].

The challenge in determining the electronic structures of the 2D BN nanosheets in the present work is compounded by additional factors, particularly the interactions between Au NPs and 2D BN nanosheets. These interactions can give rise to plasmonic effects that are one of the main factors responsible for the observed differences in electronic and optical properties of the prototype. Such plasmon-induced modulation likely accounts for the enhanced super broadband response observed in the light-induced photocurrent data.

Theoretical calculations based on density functional theory (DFT) indicate that the Schottky barrier height of Au on h-BN is generally considered around 2.0 eV. This high barrier is due to the wide bandgap of h-BN and the relatively low work function of gold. However, the exact barrier height varies, depending on other factors such as the interface structure, presence of defects, and surface reconstruction at the metal-semiconductor interface, all of which are challenging to fully characterize experimentally. A more detailed discussion of the Schottky barrier height for Au/h-BN interfaces can be found in previous reports [49]. In the present case, EDS analysis confirmed the synthesis of B-rich BN. The Schottky barrier height of Au on B-terminated h-BN has been reported to be around 0.8 eV.

**Figure 11:**
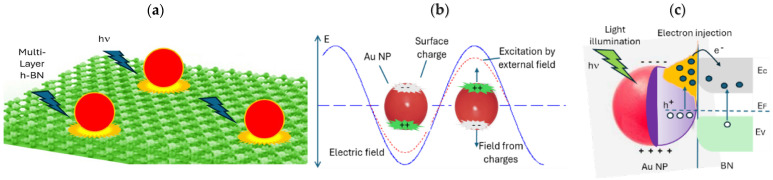
Schematic diagrams of the mechanisms of plasmonic energy transfer from Au NPs to h-BN semiconductor. (**a**) Illumination of Au NPs on multiple-layer h-BN by light with photon energy hν, initiating photon-matter interactions. (**b**) Plasmon oscillation within Au NPs upon light excitation, manifested as an external electric field (E), along with the resulting charge redistribution within the NPs. (**c**) Influence of plasmonic phenomena on charge carriers in h-BN. E_c_: the conduction band edge; E_v_: the valence band edge, and E_F_: the Fermi level.

Figure 11a shows a schematic of the Schottky barrier formed at the interface of Au NPs and h-BN semiconductor. Nonlinear I-V curves (Figure 2) confirm the presence of a Schottky-type p-n junction at the interface, where the barrier height governs charge transport. Under illumination, the photon absorption generates electron−hole pairs, initiating photocurrent generation through carrier transport and collection processes. In Figure 11b, Au nanoparticles act as plasmonic resonators. When excited by light at their plasmon resonant frequency, collective electron oscillations create intense local electromagnetic fields. This phenomenon, known as localized surface plasmon resonance (LSPR), enhances light-matter interactions and boosts photocurrent generation [50,51,52,53]. The applied electric field (E) induces charge redistribution within NPs, forming an internal dipole moment. This polarization effect is critical for plasmon-mediated energy transfer and hot carrier generation.

Figure 11c illustrates the photophysical process in the Au/h-BN heterostructure. Photons with energy hn (where h is Planck’s constant and n is frequency) excite electrons in Au NPs, generating electron-hole pairs. The holes (h^+^) remain in the Au, while the hot electrons (e^−^) are injected into the h-BN material if the energy of the excited electrons in the Au NP is high enough to overcome the energy barrier and reach the conduction band of h-BN. This directional charge transfer is enabled by the type-II band alignment at the interface [54].

The tunable bandgap and super broadband response observed in the prototype can be ascribed to the synergistic interaction between Au NP and 2D BN nanosheets, including the plasmonic enhancement of light absorption across multiple wavelength, efficient hot carrier injection via Schottky junctions, p-n heterojunction formation at the interface, and 2D confinement effects in h-BN nanosheets. Nanoplasmonic systems, characterized by enhanced local field, resonant behavior, sub-wavelength light confinement, and slow group velocity, have revolutionized optoelectronic applications. Key mechanisms include light trapping, plasmon-induced resonance energy transfer (PIRET), and hot electron injection [55]. These processes collectively improve device efficiency by increasing carrier generation and reducing recombination losses.

The significant photoresponse degradation at 400 °C is therefore attributed to composite-specific failures (agglomeration and/or oxidation) rather than hBN’s intrinsic instability. While direct high-temperature SEM/EDS data are currently unavailable, this mechanism aligns with established literature on metal-2D material nanocomposites and the known thermal limitations of Au nanoparticles in heterostructures [56,57,58]. Moving forward, high-temperature in situ characterization (e.g., dynamic SEM/EDS under controlled atmospheres) will be critical to validate these mechanisms and guide the development of more thermally robust plasmonic photodetectors.

### 3.2. First Principles Calculations

To understand the interactions between gold nanoparticles with the h-BN substrate, first-principles calculations were carried out using the Quantum Espresso (QE) package [59], which is based on density functional theory (DFT) with a plane-wave basis set [60]. The exchange correlation functional is a generalized gradient approximation (GGA) of the Perdew–Burke–Ernzerhof (PBE) form [61]. For a real multi-layer h-BN nanosheet, the situation is much more complex due to varied stacking arrangements. For simplicity, our simulations only considered a single h-BN monolayer.

In h-BN monolayer calculations, the projected augmented wave (PAW) method has been adopted to describe the pseudopotentials, and the valence configurations for construction of the PAW potentials are B (2s^2^2p^1^), N (2s^2^2p^3^), and Au (5d^10^6s^1^). Additionally, the DFT-D3 van der Waals (vdW) correction proposed by Grimme is utilized to describe the weak vdW interactions [62], which improves the accuracy of structural and electronic property calculations. It is well-known that DFT is notorious for underestimating bandgaps [63,64]. Hence, the Heyd–Scuseria–Ernzerhof (HSE06) hybrid density functional with 25% Hartree–Fock exchange energy has been used to obtain more accurate electronic properties [65].

For monolayers, an energy cut-off of 800 eV, energy convergence tolerance of 1.0 × 10^−8^ eV, convergence thresholds for atomic force of 10^−2^ eV/Å, and a Monkhorst-Pack k-point grid of 7 × 7 × 1 for a 1 × 1 unit for BN monolayers and 2 × 2 units for BN systems are found to be sufficient for geometrical optimization and electronic structure calculations. For vacancies, an energy cut-off of 500 eV, energy convergence tolerance of 1.0 × 10^−6^ eV, and 5 × 5 × 1 k-point grid were used for 2 × 2 units of BN. A thickness of 20 Å of vacuum between neighboring layers has been adopted to avoid their interactions. All the structures were fully relaxed until the Hellmann–Feynman force on each atom was <0.01 eV/Å, leading to a bond length of 1.45 Å.

**Figure 12:**
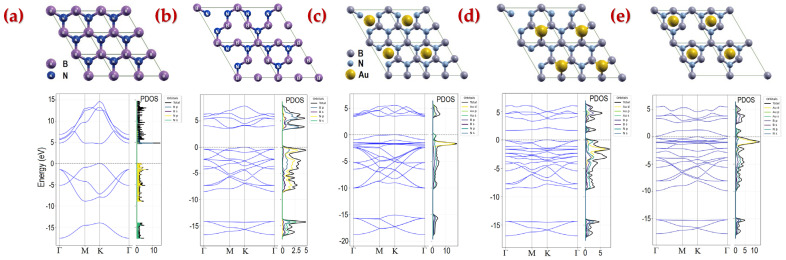
Relaxed atomistic schematics and calculated band structures and projected density of states (PDOS) of (**a**) pristine h-BN monolayer, (**b**) N-deficient h-BN, (**c**) pristine h-BN monolayer with Au atom located at the center of the hexagon, (**d**) N-deficient h-BN with Au atom located above nitrogen atom with a vertical distance of 1.72 Å, and (**e**) N-deficient h-BN with Au atom located at the center of the hexagon with a vertical distance of 2.10 Å.

The bandgap of the pristine h-BN monolayer is calculated to be 4.676 eV, as shown in Figure 12a. This value is a well-known underestimation typical of DFT simulations when compared to the experimentally reported bandgap of approximately 6 eV [66,67], even with the inclusion of correction methods such as HSE06. For an N-deficient h-BN monolayer, the bandgap decreases to 3.557 eV (Figure 12b). The PDOS (projected density of states) curves show how much each atom or atomic orbital contributes to the total density of states (DOS) at various energy levels.

When a gold atom is attached to the pristine h-BN, the band gap further reduces to 3.448 eV (Figure 12c). PDOS analysis shows the appearance of new states in the energy gap due to the Au atom. Notably, for N-deficient h-BN with a gold atom, the calculated bandgaps drop significantly to 1.651 eV (Figure 12d) and 0.115 eV (Figure 12e), depending on the Au atom’s position. While the simulations were conducted on simplified material models, the results consistently indicate that the defects in h-BN nanosheet and gold functionalization lead to substantial band gap narrowing, which supports the broadband photodetector operation.

While DFT simulations provide a quantitative explanation of the modifications to the electronic bandgap caused by a gold atom on the h-BN surface, classical plasmon resonance in gold nanoparticles on a substrate can be simulated using the MNPBEM toolbox developed by Hohenester and Trügler, which solves Maxwell’s equations for metal nanoparticles using the boundary element method [68]. Gold nanoparticles, modeled for example as nanodisks [69,70], can be further optimized in terms of size, shape, aspect ratio, and interparticle spacing, using the dielectric functions of both h-BN and gold.

## 4. Synthesis of 2D BN Nanosheets and Au NPs

The present work utilizes the same nanostructured BN materials synthesized years ago, which have since been used to develop several visible-blind deep UV photodetectors [6,20]. Re-testing exhibited reliable operation with sustained accuracy over time, and minimal performance degradation in sensitivity, stability, and response time. In the present work, we functionalized the samples with Au nanoparticles, resulting in an excellent response not only in visible and infrared regions but also with an improved response strength in the UV region compared to the original BN-based detector.

A detailed discussion of this deposition technique has been reported in our previous work [71,72]. Briefly, a high-power, pulsed CO_2_ laser deposition technique is used to synthesize atomically thin 2D BN sheets. This technique leverages the thermal energy of short laser pulses, where heat-driven mechanical exfoliation serves as the dominant mechanism in the formation of 2D crystalline BN sheets. As a result, high-purity, atomically-thin, crystalline BN sheets are obtained.

A pulsed CO_2_ laser (pulse width: 1~2 µs; repeatable rate: 5~10 Hz; pulse energy: 3~5 J), is focused using a ZnSe lens with a 30 cm focal length and directed onto a rotating pyrolytic hexagonal BN target (99.99% purity, B/N ratio~1, density~1.94 g/cc), spinning at approximately 200 rpm. The laser beam forms a ~2 mm diameter spot on the target surface, yielding a power density of approximately 2 × 10^8^ W/cm^2^ per pulse. Si wafers, used as substrates, are placed 4 cm away from the target, with the substrate temperature controlled at 350–400 °C. The deposition process lasts for 10 min, producing BN samples with a thickness of approximately 1 µm.

Au NPs are fabricated by using RF magnetron sputtering. The substrate-to-target distance is maintained at 10 cm, and deposition is conducted at room temperature with an RF power of 100 W for less than 5 s. The chamber’s base pressure is maintained at 10^−5^ Torr, while the working pressure, regulated by argon gas, is kept between 8 and 10 mTorr. Finally, the sample undergoes annealing in air at 800 °C for 30 min to enhance Au NPs-BN integration.

## 5. Conclusions

A high-performance photodetector based on Au nanoparticle-functionalized 2D BN nanosheets has been developed, demonstrating exceptional responsivity and ultra-broad spectral sensitivity. Under 250 nm illumination, the device exhibits a responsivity of 45 mA/W in photovoltaic mode (zero bias), which increases to 154 mA/W at 0.5 V and reaches a maximum of 1015 mA/W at a 2.5 V bias. The response time is approximately 0.5 ms. Although the characterization range was limited by light source availability, the device responds to a super-broad spectral range from 300 nm to 5.9 µm, covering deep ultraviolet (DUV) to mid-infrared (MIR) wavelengths.

The device operates effectively in both self-powered photovoltaic and photoconductive modes. In photovoltaic mode, it demonstrates high sensitivity without requiring external power, making it ideal for low-energy applications. When operating in photoconductive mode, the application of a bias voltage dramatically enhances photocurrent generation. For example, red light (650 nm) induces photocurrents approximately 7, 65, and 100 times greater at 0.5 V, 1.5 V, and 2.5 V bias, respectively, compared to unbiased conditions.

The tunable bandgap and super broad band response are directly linked to the synergistic interaction between the Au NPs and 2D BN nanosheets, arising from the heterostructure, and p-n heterojunction formation. The fabricated photodetector has demonstrated remarkable advantages, including fast response, high photocurrent, stable baseline, and repeatability across a wide spectral range at operating temperatures up to 100 °C. Thermal stability is another key strength. The device maintains a stable baseline and excellent repeatability up to 100 °C. For instance, under blue light, the photocurrent decreases from 250 nA at 40 °C to 170 nA at 100 °C, while the background noise remains largely unchanged, keeping the signal-to-noise ratio around 30, indicating robust performance at elevated temperatures.

The remarkable performance stems from several synergistic effects. The built-in electric fields at the interface between Au nanoparticles and BN nanosheets enable efficient separation of photogenerated electron–hole pairs. The localized surface plasmon resonance (LSPR) triggered by the varying sizes, shapes, and spacing of the Au NPs contributes significantly to light absorption. The plasmonic field enhancement from the Au NPs increases the interaction of light with nearby BN molecules, boosting overall device sensitivity. Additionally, h-BN’s atomically flat and clean surface minimizes background signals and scattering noise, further improving signal clarity. The presence of hyperbolic phonon polaritons in the mid-IR range also enhances broadband optical sensing, particularly in the MIR region.

Moreover, the tunable bandgap and broadband response result from the synergistic interactions between Au NPs and 2D BN nanosheets, including heterostructure formation and p-n junction effects. These features make the photodetector not only suitable for optical communication, imaging, and environmental sensing but also particularly promising for chemical or biosensor applications due to its high sensitivity, broadband response, and operational stability under harsh conditions. In conclusion, the Au-functionalized 2D BN photodetector offers a compelling combination of broadband detection, high responsivity, rapid response, plasmon-enhanced light–matter interaction, and robust thermal performance. These characteristics make it a strong candidate for next-generation optoelectronic devices, especially in flexible, wearable, and harsh-environment applications.

## Data Availability

The original contributions presented in this study are included in the article. Further inquiries can be directed to the corresponding authors.
